# A case of cup‐like blasts associated with B‐lymphoblastic leukemia without *NPM1* and *FLT3* internal tandem duplication mutations

**DOI:** 10.1002/jha2.91

**Published:** 2020-09-08

**Authors:** Jean‐Baptiste Rieu, Lucie Coster, Jill Corre, Suzanne Tavitian, Christian Recher, Anne Huynh, Muriel Picard, Celine Derreumaux, Sarah Bertoli, Francois Vergez, Laetitia Largeaud

**Affiliations:** ^1^ Haematology Laboratory Cancer University Institute of Toulouse – Oncopole Toulouse France; ^2^ Clinical Haematology Unit Cancer University Institute of Toulouse – Oncopole Toulouse France; ^3^ Intensive Care Unit Cancer University Institute of Toulouse – Oncopole Toulouse France

The importance of flow cytometry immunophenotyping, cytogenetic and molecular analysis in diagnosis and prognosis of acute leukemia has greatly increased in recent years and tends to gradually replace morphological examination. Nevertheless, the latter still keeps a central place and should not be overlooked. Several correlations between morphological signs and cytogenetic and molecular abnormalities have been identified in acute myeloid leukemia (AML), including the well‐known “faggot cells” in acute promyelocytic leukemia with *PML – RARA* and abnormal eosinophilic bone marrow proliferation in AML with inv(16) or t(16;16); *CBFB – MYH11*. As well, at the end of the 2000s, a new distinctive morphologic sign associated with a particular phenotype, cytogenetic and molecular profile in acute leukemia, has been described and defined the so‐called cup‐like blasts [1].

These blasts with prominent nuclear invagination are strongly associated with AML with normal karyotype and nucleophosmin 1 (*NPM1*) mutation, with or without co‐occurring fms‐related tyrosine kinase 3 (*FLT3*) internal tandem duplication (ITD) mutation [1]. *NPM1* mutation is one of the most common recurrent genetic lesion in AML and is relatively specific for AML [2]. Concurrent *FLT3*‐ITD mutation is associated with higher white blood cell (WBC) count, higher percentages of bone marrow blasts, and worse prognosis [3].

The morphology of cup‐like blasts is defined by a nuclear invagination spanning at least 25% of the nuclear diameter [4]. According to different authors, a minimum of 5% [1] or 10% [4] of blasts presenting this specific imprint of the nucleus is necessary in peripheral blood and/or bone marrow to define AML with cup‐like morphology. Based on the French‐American‐British (FAB) classification, the majority of cases are associated with AML without maturation (M1), acute myelomonocytic leukemia (M4), and acute monocytic leukemia (M5) but other types of AML may be concerned [5].

A flow cytometric immunophenotyping pattern of cup‐like blasts is characterized by the lack of CD34 and HLA‐DR expression and expression of cytoplasmic myeloperoxidase [1, 2, 3, 4, 5]. Aberrant expression of B‐cell or T‐cell markers is not expected.

**FIGURE 1:**
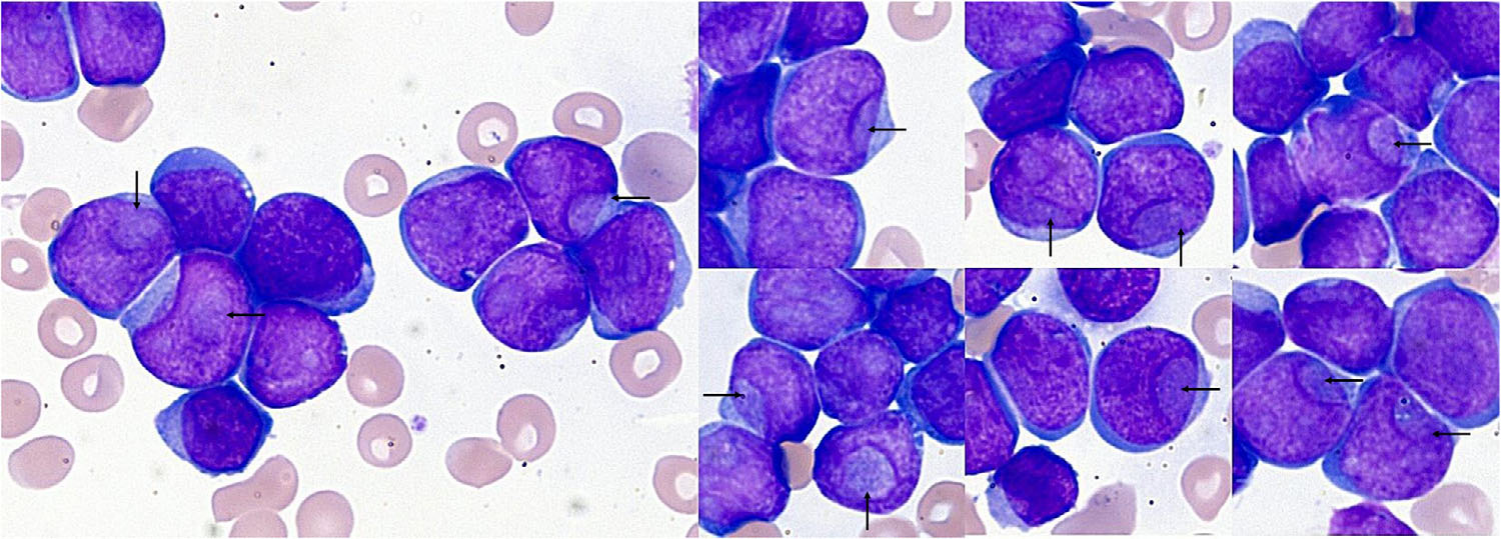
Blast cells with prominent nuclear invagination (black arrows) defining cup‐like morphology in peripheral blood smear from the patient (May‐Grünwald‐Giemsa stain, original magnification ×1000)

We report the case of a 46‐year‐old woman hospitalized for fatigue, dyspnea, night sweats, and metrorrhagia of recent onset. Eight days before her admission, she was in perfect health. Clinical examination was normal at the entrance. The blood count showed severe anemia (hemoglobin: 37 g/L), thrombocytopenia (64 × 10^9^/L), and extreme leukocytosis (617 × 10^9^/L). Blast cells represented 97% of WBC on the blood film. They were medium/high size with high nuclear‐cytoplasmic ratio, round nucleus, and basophilic nongranular cytoplasm. Approximately one‐third of these cells had prominent round and translucent invagination, which overlapped more than 25% of the nucleus and therefore met the definition of cup‐like blasts (Figure 1).

**FIGURE 2:**
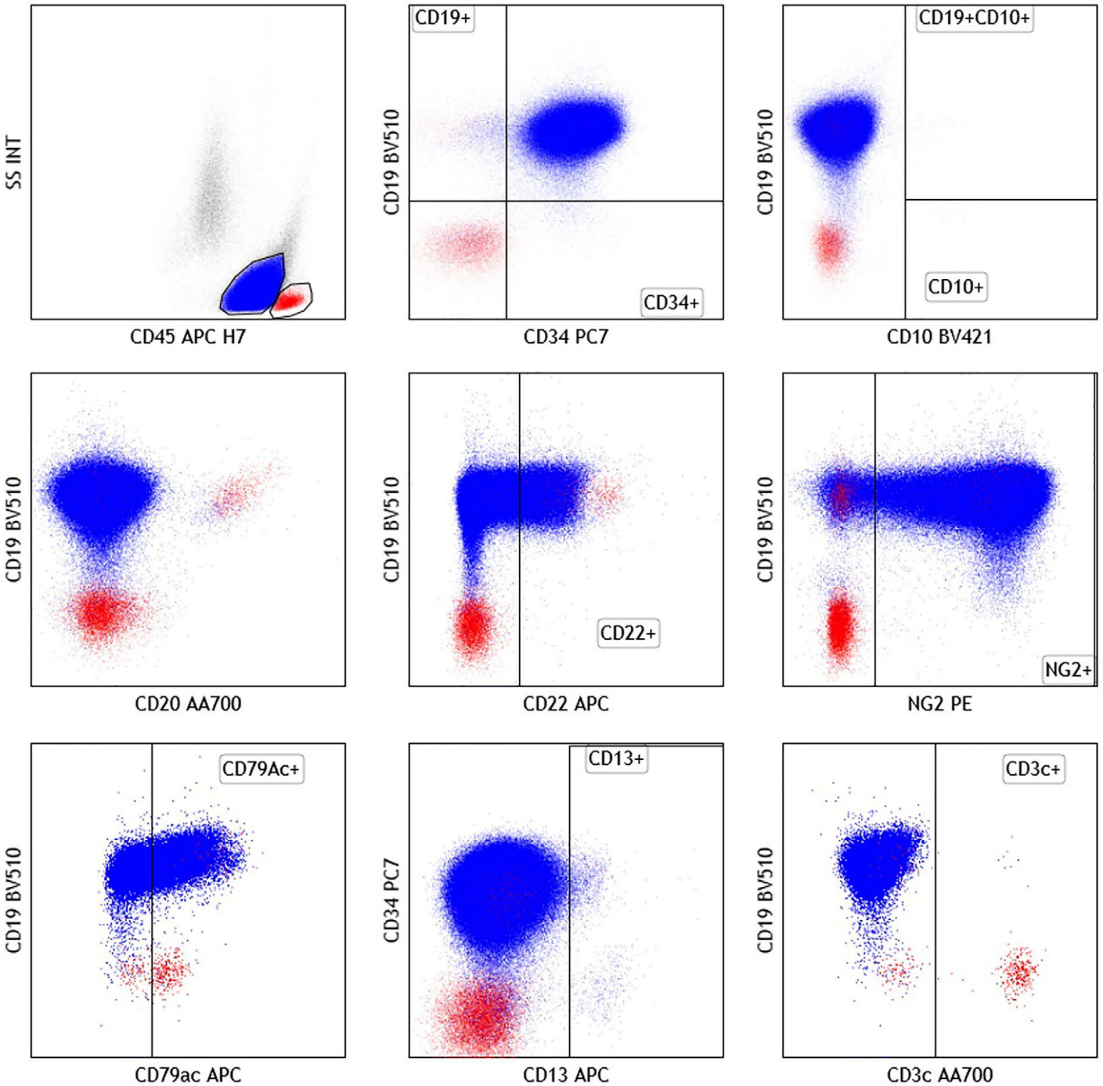
Immunophenotype of blast cells (blue) and lymphocytes (red) from the patient (10‐color flow cytometry using a Beckman Coulter Navios cytometer and analyzed with Kaluza software)

Flow cytometric immunophenotyping (Figure 2) showed strong expression of CD34, CD19, and cytoplasmic CD79a, weak CD22, and no CD10 and CD20. Cytoplasmic myeloperoxidase was not expressed, as there were other markers of the myeloid or T‐cell lineage. We also noticed the expression of NG2 commonly associated with *KMT2A* rearrangement in B‐ALL [6]. Cytogenetic analysis showed t(4;11)(q21;q23) ; *KMT2A‐AFF1*. Next‐generation sequencing revealed *KRAS* mutation (G13D, VAF 49%). No mutation was detected in *NPM1* or *FLT3*. These results converged to the diagnosis of B‐lymphoblastic leukemia (B‐ALL) with cup‐like morphology.

Given the results of the full blood count, the patient was immediately transferred to the intensive care unit (ICU). Upon arrival in ICU, she presented right hemiplegia with facial paralysis. Computed tomography (CT) scan revealed a large brain hematoma without medical or surgical possibility of treatment. Despite appropriated intensive cares, the patient's neurological condition continued to deteriorate and she died few hours later.

The strong correlation between AML with cup‐like blasts and *NPM1* or concurrent *NPM1* and *FLT3*‐ITD mutations is well recognized and reported in numerous studies [1, 2, 3, 4, 5]. But the specificity of cup‐like blasts as a predictive factor for *NPM1* and/or *FLT3*‐ITD mutations is restricted to 60% [5]. Thus, this morphologic sign is not always associated with *NPM1* and *FLT3*‐ITD mutations. Furthermore, cup‐like blasts must not be considered as a specific feature of AML. Two others B‐ALL with cup‐like blasts occurring in adult patients are reported in the literature [7, 8]. One presented with marked leukocytosis [7], but leukocyte count was not mentioned in the second [8]. *NPM1* and *FLT3* mutations were not found in either. B‐ALL with t(9;22); *BCR – ABL1* was finally diagnosed in the first case [7], while the second showed B‐ALL with near‐triploid karyotype and *TP53* and *DNMT3A* mutations [8]. Flow cytometry immunophenotyping and/or immunohistochemical studies showed expression of CD19, CD10, CD22, TdT, and CD34, without myeloid or T‐cell lineage markers [7, 8]. Complete remission after induction was achieved in both cases [7, 8]. One similar case was reported in a 7‐year‐old girl and was associated with normal karyotype and *IKZF*1 deletion [9]. A subsequent retrospective study of 425 B‐ALL patients aged 10 days to 19 years tends to confirm the correlation between cup‐like blasts and *IKZF*1 deletion in pediatric B‐ALL [10]

Blasts with cup‐like morphology are commonly described in AML with *NPM1* and/or *FLT3* mutations, but these rare presentations of B‐ALL remind us that the presence of cup‐like blasts, even in high proportion, is not a morphologic argument for assigning blasts in the myeloid lineage.

According to the World Health Organisation (WHO) classification [2], acute leukemias with *KMT2A‐AFF1*‐rearranged are usually associated with high WBC count and dysmorphic blast population with a variable proportion of blasts meeting the morphologic criteria of monoblasts and others of lymphoblasts. Most cases are B‐ALL, but AML and mixed‐phenotype are also reported [2]. We retrospectively reviewed bone marrow smears from five adults B‐ALL with *KMT2A‐AFF1*‐rearranged diagnosed in our laboratory, and one presented a cup‐like morphology according the threshold of 10% (one had 3% of cup‐like blasts, the others 0%).

Cases of B‐ALL in adult patients with cup‐like blasts are exceptionally described. Given our results and the heterogeneity of the cases reported (Table 1), it is to date not possible to correlate this morphologic sign with *KMT2A‐AFF1* or other cytogenetic or molecular signature in adult B‐ALL. But this finding might be underestimated. A retrospective study with further investigations in adults B‐ALL, as it was done in pediatric B‐ALL [10], be needed to define whether or not cup‐like blasts are associated with cytogenetic/molecular abnormalities and might become a relevant morphologic sign in the prognosis in adult B‐ALL.

**TABLE 1:** Immunophenotypic, cytogenetic and molecular genetic features of B‐ALL with cup‐like morphology reported in literature

Reference	Age (years)	Sex	Immunophenotype	Conventional cytogenetic study	Other genetic abnormalities
Rieu et al. [11]	46	F	CD19+ CD10‐ CD34+	46,XX,t(4;11)(q21;q23) ; *KMT2A‐AFF1*	*KRAS*
Mehtap et al. [7]	41	M	CD19+ CD10+ CD34+	46,XY,t(9;22)(q34.1;q11.2) ; *BCR‐ABL1*	‐
Wang et al. [8]	70	M	CD19+ CD10+ CD34+	72∼80,XXY,−3,+4,+6,−7,+8,+8,+8,+8,−9,+10,+11,−12,−14,−16,+18,+21,+22,+3∼5mar[cp5]	*TP53* *DNMT3A*
Richardson et al. [9]	7	F	CD19+ CD10+ CD34+	46,XX	*IKZF1*
